# Summer-form susceptible, winter-form resistant: differential susceptibility of *Cacopsylla chinensis* nymphs to *Beauveria bassiana*

**DOI:** 10.3389/fcimb.2026.1924929

**Published:** 2026-08-19

**Authors:** Jiayue Ji, Chendong Yue, Zengli Hu, Bingnan Chen, Longlong Zhao

**Affiliations:** 1 Institute of Pomology, Shanxi Agricultural University, Jinzhong, China; 2 College of Plant Protection, Shanxi Agricultural University, Jinzhong, China

**Keywords:** *Beauveria bassiana*, biological control, *Cacopsylla chinensis*, immune response, metabolic regulation, seasonal polyphenism

## Abstract

**Introduction:**

The pear psylla *Cacopsylla chinensis* is a destructive pear pest whose nymphs exhibit seasonal polymorphism between summer-form and winter-form morphs. Whether the two morphs differ in susceptibility to *Beauveria bassiana* and the underlying mechanisms have not been characterized.

**Methods:**

Bioassays at three spore concentrations (2.5×10^6^, 2.5×10^7^, and 2.5×10^8^ spores/mL) were conducted to compare fungal virulence against fourth-instar nymphs of both morphs. Hemocyte counts and transcriptome sequencing were performed at 24 h and 36 h post-infection, respectively. qRT-PCR and enzymatic activity assays were used to further validate immune-related gene expression and enzyme activities.

**Results:**

B. bassiana exhibited significantly higher virulence against summer-form nymphs than winter-form nymphs across all concentrations; at the highest concentration, only 8% of summer-form survived to day 5, compared with approximately 40% survival in winter-form, representing an approximately 79-fold difference in LC_50_ between the two morphs. Transcriptome analysis revealed that winter-form nymphs triggered a much broader transcriptional response with 1,673 differentially expressed genes (1,344 upregulated), accompanied by coordinated induction of immune genes including phenoloxidase, lysozyme and catalase, alongside full activation of energy metabolism pathways such as oxidative phosphorylation and the TCA cycle. In contrast, summer-form nymphs showed weak immune and metabolic activation. qRT-PCR and enzyme activity assays further verified that key immune-related transcripts and enzyme activities were markedly more induced in winter-form nymphs.

**Discussion:**

These findings demonstrate that winter-form nymphs possess a more robust immune-metabolic defense network against *B. bassiana*, whereas summer-form nymphs are immune-suppressed and highly susceptible, revealing the molecular basis of morph-specific pathogen resistance shaped by seasonal polyphenism and providing a mechanistic basis for seasonally optimized biocontrol strategies.

## 1 Introduction

China is the world’s largest pear producer, with United Nations Food and Agriculture Organization (FAO) data showing that from 2019 to 2023, China’s pear planting area was approximately 900,000 hectares, with an annual average yield exceeding 18 million tons, ranking first globally. The healthy development of the pear industry plays a pivotal role in ensuring fruit supply and increasing farmers’ income. Among pear tree pest groups, *Cacopsylla chinensis* is the most closely associated with pear trees, exhibiting particularly prominent damage (Chen et al., 2018). In northern pear-producing regions, *C. chinensis* causes an average annual pear yield loss of approximately 10%, with control costs ranging from 6,000–10,500 RMB per hectare, making it an important target for pest management. *C. chinensis* is widely distributed across major pear-growing regions in China, adapting to primary cultivated pear varieties such as Chinese white pear and sand pear. *C. chinensis* feeds on pear fruit, phloem, and leaf sap, causing leaf drop, tree vigor decline, and reduced fruit quality (Ge et al., 2020). Additionally, honeydew secreted by nymphs interferes with natural enemy predation and promotes sooty mold growth, further impeding photosynthesis and reducing tree vigor, fruit yield, and marketability (Ge et al., 2020). *C. chinensis* exhibits seasonal plasticity in phenotype, divided into summer-form and winter-form morphs. The summer-form primarily occurs during the pear tree growth period, while the winter-form persists through the pear tree dormancy, budding, flowering, and leaf expansion stages (Wei et al., 2020).

In the management of *C. chinensis*, most pear-producing regions still rely on chemical control. High-intensity pesticide application has led to the development of resistance in *C. chinensis* to multiple insecticides including pyrethroids, organophosphates, neonicotinoids, and spirotetramat, accompanied by negative impacts such as reduced natural enemy populations, environmental pollution, and food safety concerns (Wang et al., 2026). Consequently, control costs have increased while control difficulty has escalated. Therefore, developing green and safe control measures has become a key research focus. Investigations indicate that predatory natural enemies of *C. chinensis* in pear orchards are mostly generalist predators, commonly including ladybugs, lacewings, and minute pirate bugs, which exhibit low selectivity and temporal synchrony (Ge et al., 2020). Among parasitic natural enemies, *Psylladintus insidiosus* has certain development and utilization potential, but its industrialization level is currently low, and related application technologies remain immature (Zhao et al., 2020). Among physical control measures, sticky traps are mainly used for monitoring and trapping adults, but their control efficacy is limited with low ecological benefits. *Beauveria bassiana* is a broad-spectrum entomopathogenic fungus widely distributed in natural environments, capable of infecting insects through cuticular contact, oral ingestion, and other routes (Ortiz-Urquiza and Keyhani, 2016). As a biocontrol microorganism with significant application potential, *B. bassiana* offers advantages including good environmental compatibility and the ability to form secondary epidemics in pest populations (Mascarin and Jaronski, 2016; Panwar and Szczepaniec, 2024). It has been widely applied in green control of agricultural and forestry pests, with commercial formulations showing high field efficacy against piercing-sucking pests such as aphids, whiteflies, and spotted lanternflies, playing important roles in reducing chemical pesticide use and ensuring agricultural product quality and safety (Clifton et al., 2020; Mascarin and Jaronski, 2016; Wang et al., 2004, 2021).

The response mechanisms of insects to pathogens are crucial factors affecting control efficacy (Zhang et al., 2024). Clarifying the defense systems of pests helps enhance pest control efficiency. Existing research indicates that insects possess comprehensive immune response systems against pathogen infection, including hemocyte-mediated clearance of pathogens invading the hemocoel through phagocytosis, encapsulation, and nodule formation, as well as defense mechanisms relying on prophenoloxidase activation cascades, antimicrobial peptide synthesis, and reactive oxygen species scavenging systems (Sheehan et al., 2018; Valanne et al., 2011). Different developmental stages exhibit variations in body structure, physiological status, activity levels, and stress responses, all of which can influence the control efficacy of pathogens (Sedighi et al., 2013; Sharififard et al., 2014). Our previous studies demonstrated that *B. bassiana* has infective capabilities against *C. chinensis* adults (Ji et al., 2025). As an important developmental stage, nymphs compared to adults exhibit weaker activity, relatively lower insecticide resistance, more concentrated distribution, and synchronized occurrence (especially first-generation nymphs), making them the optimal window for control (Ji and Zhao, 2022). However, whether *B. bassiana* has control potential against *C. chinensis* nymphs, whether different seasonal morphs exhibit differential resistance to this fungus, and what the underlying molecular regulatory mechanisms are, have not been investigated. Therefore, this study investigated the virulence of *B. bassiana* against different seasonal morphs of *C. chinensis* nymphs and analyzed the resistance difference mechanisms using transcriptome sequencing technology, aiming to provide theoretical support for developing precise biocontrol strategies against *C. chinensis* nymphs and optimizing field application scenarios of *B. bassiana*.

## 2 Materials and methods

### 2.1 Insect rearing and fungal culture

Eggs of *C. chinensis* were originally collected from the pear orchard at the Pomology Institute, Shanxi Agricultural University (37°20′35″ N, 112°29′32″ E). Summer-form nymphs were obtained under rearing conditions of 25 °C and 12L:12D photoperiod, fed with Yuluxiang pear branches. Winter-form nymphs were induced in the artificial climate chamber at 20 °C with 8L:16D photoperiod, also fed with Yuluxiang pear branches. During experiments, all rearing conditions were maintained at 25 °C, 60–80% relative humidity, and 12L:12D photoperiod. The fungal inoculum used *B. bassiana* strain GSSBb1901 isolated and preserved by our laboratory. After culturing on PDA plates at 25 °C and 60–80% relative humidity for 15 days, spores were washed with PBS solution containing 0.05% Tween-80 and suspended to the required concentration.

### 2.2 Bioassays and hemocyte counting

*B. bassiana* spore suspensions were prepared at concentrations of 2.5×10^6^, 2.5×10^7^, and 2.5×10^8^ spores/mL. Healthy 4th-instar nymphs of both seasonal morphs were selected, and 1 μL of the corresponding spore suspension was applied onto the dorsal junction of the thorax of each nymph using a micropipette, then gently spread to facilitate drying. Ten nymphs were placed in one Petri dish to reduce physical contact and mutual scraping that might wipe off the fungal inoculum. Each concentration treatment consisted of 10 independent Petri dish replicates, with a total of 100 C*. chinensis* individuals tested per concentration; PBS inoculation served as the blank control. Culture dishes containing nymphs were transferred to a constant temperature incubator set at 25 °C, 75% relative humidity, and a 12L:12D photoperiod, and the number of dead nymphs was counted daily. No censoring occurred during the 5-day observation period, as all nymphs were accounted for at each time point. Mortality was assessed based on unambiguous objective criteria: complete immobility and desiccation of the nymph body, minimizing the potential for observer bias.

To determine the hemocyte concentration, 4th-instar summer-form and winter-form *C. chinensis* nymphs were infected with *B. bassiana* spore suspension at 2.5×10^7^ spores/mL, with 1 μL of spore suspension dropped onto each nymph’s surface. Hemolymph was collected by puncturing the abdomen of each nymph with an insect needle at 24 h after infection. 0.05 μL of fresh hemolymph was diluted with 10 μL ice-cold PBS and counted using the hemocytometer. Two nymphs were used for each sample. One sample was counted twice. Each treatment was replicated seven times.

### 2.3 Sample preparation and transcriptome sequencing

Healthy 4th-instar nymphs of both morphs were collected separately. Each insect was infected with 1 μL of *B. bassiana* spore suspension at 2.5×10^7^ spores/mL, then cultured in a climate-controlled chamber. After 36 hours, insects were collected into 1.5 mL centrifuge tubes, rapidly frozen in liquid nitrogen, and subsequently stored in liquid nitrogen for transcriptome sequencing. Individuals treated with equal volume PBS served as controls, with 0.1 g of material collected per treatment, and each treatment was repeated three times. Total RNA was extracted using the TRIzol method, with RNA concentration and purity detected using Nanodrop 2000 spectrophotometer and RNA integrity assessed using Agilent 2100. Subsequently, qualified samples underwent a series of processes including enrichment, fragmentation, cDNA synthesis and purification to ultimately obtain cDNA libraries. After library quality inspection, sequencing was finally performed on an Illumina NovaSeq 6000 platform. The above sequencing was performed at Biomarker Technologies (Beijing, China).

### 2.4 Transcriptome data analysis

After sequencing data generation, clean data were assembled to obtain the unigene library for subsequent analysis. DIAMOND software was used to annotate unigene sequences against multiple databases including NR, Swiss-Prot, COG, KOG, KEGG, and eggNOG database. KOBAS was used to obtain the KEGG Orthology annotations of unigenes in the KEGG database. InterProScan was employed to analyze the Gene Ontology annotations of novel genes by leveraging the integrated databases within InterPro. The amino acid sequences of unigenes were predicted using TransDecoder, and subsequently aligned against the Pfam database using HMMER. In this study, unigene annotations were obtained by applying a BLAST E-value threshold of ≤ 1e^-5^ and an HMMER E-value threshold of ≤ 1e^-10^. Bowtie was used to align reads to the unigene library, combined with RSEM software to calculate gene expression levels, using FPKM (Fragments Per Kilobase of transcript per Million mapped reads) as the gene expression abundance normalization indicator. Spearman correlation coefficient was used to assess biological replicate correlations, combined with PCA to verify between-group and within-group sample dispersion. DESeq2 software was used for differential analysis of different treatment group data, with the Benjamini–Hochberg procedure applied to control the false discovery rate (FDR < 0.05), and |log_2_Fold Change| ≥ 1 as the screening criteria for differentially expressed genes (DEGs). All 12 samples were processed in a single sequencing batch; therefore, no batch correction was required. GO functional enrichment and KEGG pathway enrichment analyses were performed on the screened DEGs.

### 2.5 Quantitative real-time PCR analysis

Twelve DEGs were selected for qRT-PCR validation, including six randomly selected transcripts from the initial transcriptome analysis and six immune-related genes (*carboxylesterase*, *CarE*; *superoxide dismutase*, *SOD*; *cytochrome P450-9*, *CYP450-9*; *catalase*, *CAT*; *glutathione S-transferase*, *GST*; *phenoloxidase-2*, *PO-2*) selected based on their relevance to immune defense and detoxification. Reactions were performed on a CFX96 Touch system (Bio-Rad, Hercules, CA, USA) with SYBR qPCR Master Mix (Tolo Biotech, Shanghai, China) per the manufacturer’s instructions. Primer sequences are detailed in **Supplementary Table 1**. The internal reference gene *rPL45* was used for normalization, and relative expression levels were computed using the 2^-ΔΔCt^ method (Ji et al., 2025).

### 2.6 Enzymatic activity assays

The treatment and sampling time points of nymphs for enzymatic activity assays were the same as those for transcriptome sequencing. The activities of SOD, CAT, GST, and PO were measured in each treatment group. Four samples were collected per treatment, with approximately 70 nymphs per sample. For each sample, approximately 70 nymphs were collected into a 1.5 mL centrifuge tube and homogenized on ice using a disposable tissue grinding pestle in the extraction buffer provided with each commercial kit. The homogenate was centrifuged at 12,000 × g for 10 min at 4 °C, and the supernatant was used for subsequent assays. Due to the small body size of C. chinensis nymphs, whole-body homogenates were used for all enzyme activity measurements. Enzyme activities of PO, SOD, GST, and CAT were quantified using commercial assay kits (PO Activity Assay Kit, G0146W; SOD Assay Kit, WST-8 method, G0101W48; GST Assay Kit, G0208F; CAT Assay Kit, visible colorimetric method, G0105W48; Suzhou Geruisi Biotechnology Co., Ltd). Total protein content was quantified using a BCA Protein Assay Kit (G0420W, Suzhou Geruisi Biotechnology Co., Ltd) for normalization of enzyme activities. All assays were performed according to the manufacturer’s instructions.

### 2.7 Statistical analysis

Statistical analysis was performed using GraphPad Prism version 9.0. Survival curves were calculated using the Kaplan-Meier method, with differences evaluated using log-rank (Mantel-Cox) test (*p* < 0.0001). Significance testing employed unpaired two-tailed t-test (*p* < 0.05). Median lethal concentration (LC_50_) values at 5 days post-infection were calculated by probit analysis using SPSS version 21.0 software, with 95% confidence intervals for each morph.

## 3 Results

### 3.1 Differential virulence of *B. bassiana* against two seasonal morphs

**Figure 1:**
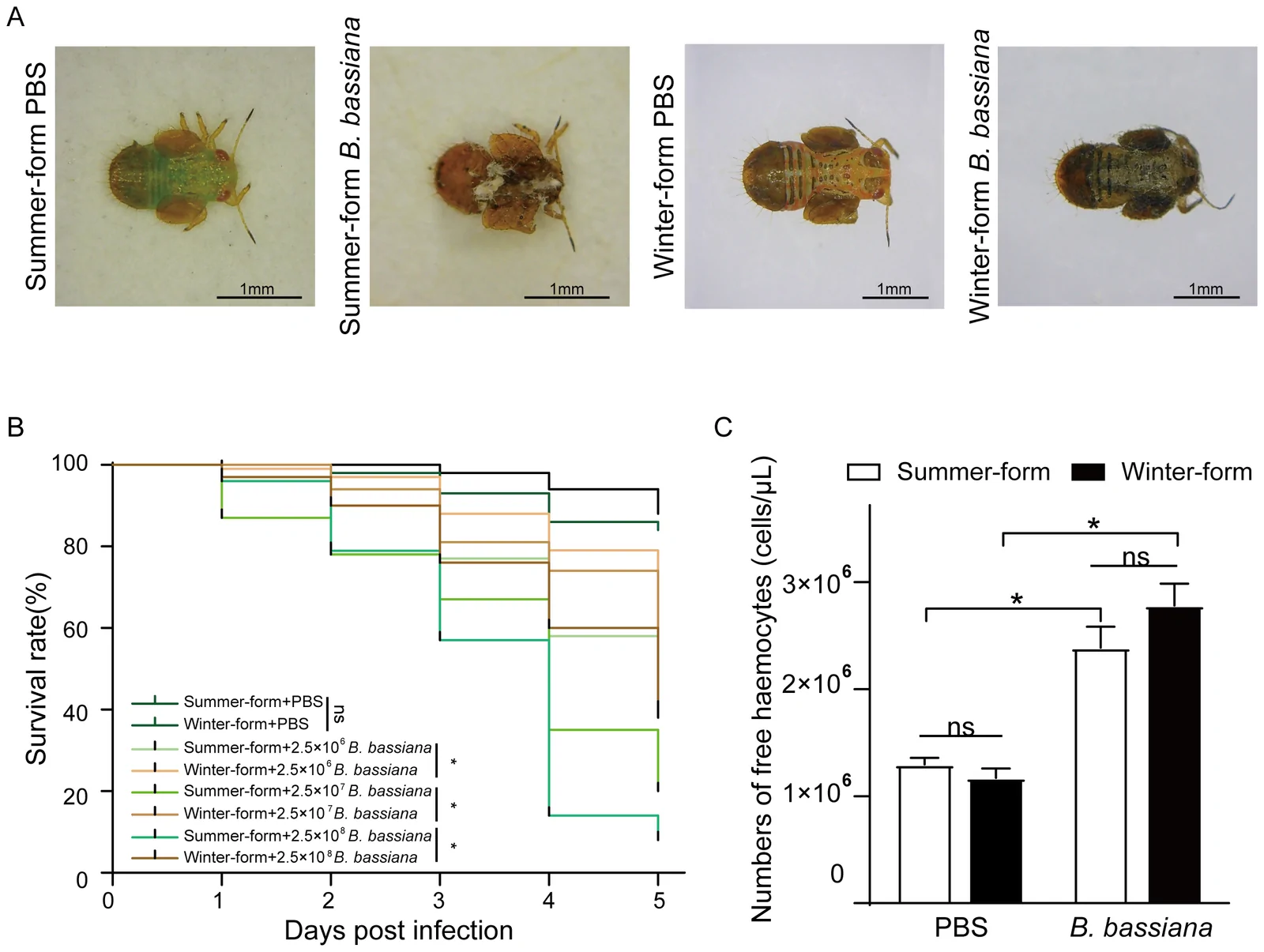
Phenotypic changes and virulence of *B. bassiana* against two seasonal morphs of *C. chinensis* nymphs. **(A)** Morphological comparison of nymphs before and after *B. bassiana* infection. **(B)** Survival curves of nymphs infected with *B. bassiana* conidia at concentrations of 2.5 × 10^6^, 2.5 × 10^7^, and 2.5 × 10^8^ conidia per individual, or treated with PBS (*n* = 100). Differences between summer-form and winter-form survival curves at each concentration were evaluated using the log-rank (Mantel–Cox) test. Asterisks indicate significant differences between the two morphs under the same treatment (*p < 0.05); ns, not significant. **(C)** Hemocyte counts in the two nymph morphs at 24 h post-infection. Asterisks indicate significant differences between PBS and *B. bassiana* treatment within each morph (unpaired two-tailed t-test: *p < 0.05); ns, not significant between morphs under the same treatment.

After inoculation with *B. bassiana*, summer-form and winter-form *C. chinensis* nymphs exhibited distinct differences in infection phenotypes. Summer-form nymphs exhibited fungal infection symptoms earlier, with body color darkening and white mycelium appearing on the intersegmental membranes, antennae, and legs, eventually leading to the entire body being covered by white mycelium, becoming shrunken and brown. In contrast, winter-form nymphs required a longer time to observe mycelial growth, indicating a significantly delayed infection process (**Figure 1A**).

To evaluate the effect of *B. bassiana* infection on the survival of summer-form and winter-form nymphs, survival rates were monitored daily over 5 days post-infection. As shown in **Figure 1B**, the survival rates of both PBS control groups remained above 84% throughout the observation period, with no significant difference detected between the summer-form and winter-form PBS groups (*df* = 1, *p* = 0.3630), indicating that the PBS treatment itself had no adverse effect on survival. Survival rates decreased with increasing concentrations of *B. bassiana*. At 5 days post-infection, in the low-concentration group (2.5×10^6^ conidia/mL), the survival rate of summer-form insects was 38%, which was significantly lower than that of winter-form insects (60%) (*df* = 1, *p* = 0.0008); in the medium-concentration group (2.5×10^7^ conidia/mL), the survival rate of summer-form insects was 20%, which was significantly lower than that of winter-form insects (53%) (*df* = 1, *p* < 0.0001); in the high-concentration group (2.5×10^8^ conidia/mL), the survival rate of summer-form insects was 8%, which was significantly lower than that of winter-form insects (40%) (*df* = 1, *p* < 0.0001) (**Figure 1B**). To further quantify the virulence difference between the two morphs, median lethal concentration (LC_50_) values were calculated by probit analysis based on mortality at 5 days post-infection. The LC_50_ for summer-form nymphs was 1.58×10^6^ spores/mL (95% CI: 2.70×10^5^ - 3.92×10^6^), whereas the LC_50_ for winter-form nymphs was 1.25×10^8^ spores/mL (95% CI: 3.40×10^7^ - 7.03×10^9^), representing an approximately 79-fold difference in susceptibility between the two morphs.

At 24 h post-infection with *B. bassiana*, hemocyte concentrations increased significantly in both morphs (summer-form: *df* = 12, *p* = 0.002; winter-form: *df* = 12, *p* < 0.001). Infected winter-form *C. chinensis* exhibited a hemocyte concentration of approximately 2.8×10^6^ cells/μL, higher than that of infected summer-form individuals (approximately 2.4×10^6^ cells/μL); however, the difference between the two morphs was not statistically significant (*df* = 12, *p* = 0.2688) (**Figure 1C**).

### 3.2 Transcriptome data overview

A total of 225.55 Gb of clean data was generated from 12 samples, with an average of approximately 7.5 Gb per sample. The Q30 percentage ranged from 92.35% to 93.90% across all samples, indicating high sequencing quality (**Supplementary Table 2**). Final assembly yielded 47,465 unigenes with an N50 of 2,003. Among eight databases (NR, Swiss-Prot, KEGG, COG, KOG, eggNOG, Pfam, and GO), 7,511 unigenes were annotated by at least one database. NR annotation showed that 51.6% of the sequences aligned to *Diaphorina citri* (**Supplementary Figures 1A, B**). After B. bassiana infection, winter-form nymphs produced 1,673 DEGs, more than the 1,203 DEGs produced by summer-form nymphs. Both morphs showed more upregulated genes than downregulated genes, with summer-form nymphs having 822 upregulated and 381 downregulated genes, and winter-form nymphs having 1,344 upregulated and 329 downregulated genes (**Supplementary Figure 1C**). qRT-PCR analysis confirmed that the expression trends of six randomly selected transcripts were consistent with the transcriptome sequencing results, verifying the reliability of the transcriptome data (**Supplementary Figure 2**).

### 3.3 Functional classification and enrichment analysis of DEGs

GO functional classification of DEGs showed that DEGs from both morphs were divided into three major categories: biological process, cellular component, and molecular function. In the biological process category, DEGs from both morphs were mainly classified into cellular process, metabolic process, biological regulation, and response to stimulus. In the molecular function category, DEGs were mainly classified into binding and catalytic activity (**Supplementary Figure 3**). KEGG functional classification of DEGs showed that DEGs from both morphs were divided into five categories: cellular processes, environmental information processing, genetic information processing, metabolism, and organismal systems. The most annotated DEGs were in the ribosome pathway, followed by protein processing in endoplasmic reticulum. Winter-form DEGs had the most annotated genes in oxidative phosphorylation, whereas this pathway was not ranked highly in summer-form annotations (**Supplementary Figure 4**).

**Figure 2:**
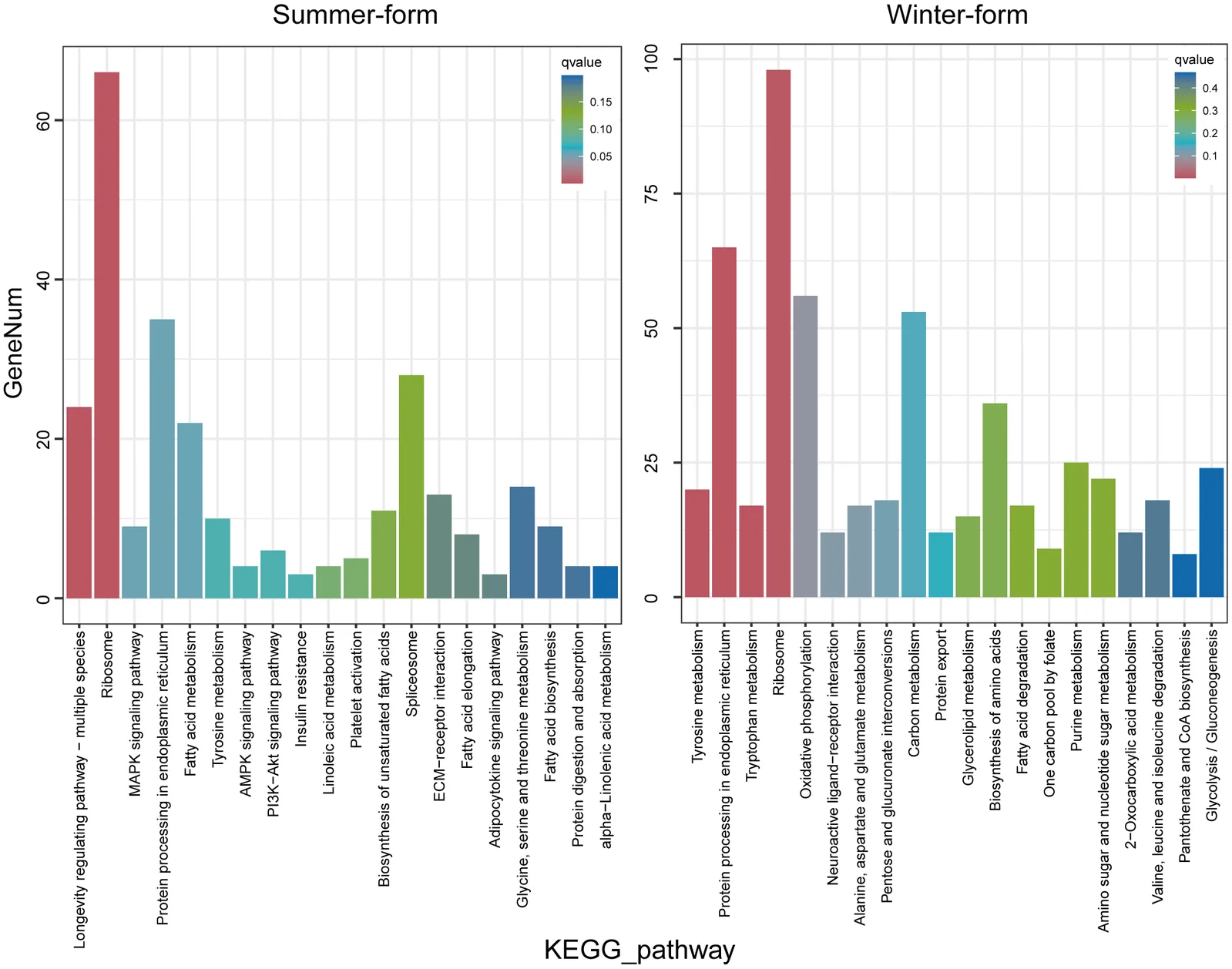
KEGG enrichment analysis of DEGs in summer-form and winter-form *C. chinensis* nymphs after *B. bassiana* infection.

KEGG enrichment analysis of DEGs showed that in summer-form samples, ribosome had the highest number of enriched genes and significance, followed by protein processing in endoplasmic reticulum and spliceosome. Additionally, longevity regulating pathway - multiple species, fatty acid metabolism, MAPK signaling pathway, AMPK signaling pathway, PI3K-Akt signaling pathway, and other signal transduction and metabolism-related pathways were significantly enriched, while biosynthesis of unsaturated fatty acids and alpha-linolenic acid metabolism pathways had fewer enriched genes and lower significance. In winter-form samples, ribosome and protein processing in endoplasmic reticulum still had the highest number of enriched genes and significance, with oxidative phosphorylation and carbon metabolism significantly enriched and having higher gene numbers. Additionally, biosynthesis of amino acids, purine metabolism, glycolysis/gluconeogenesis, tyrosine metabolism, and other amino acid and carbohydrate metabolism-related pathways were significantly enriched, with overall energy metabolism-related pathways showing higher enrichment degrees and gene numbers than in summer-form (**Figure 2**).

### 3.4 Response differences in key pathway genes between two morphs

**Figure 3:**
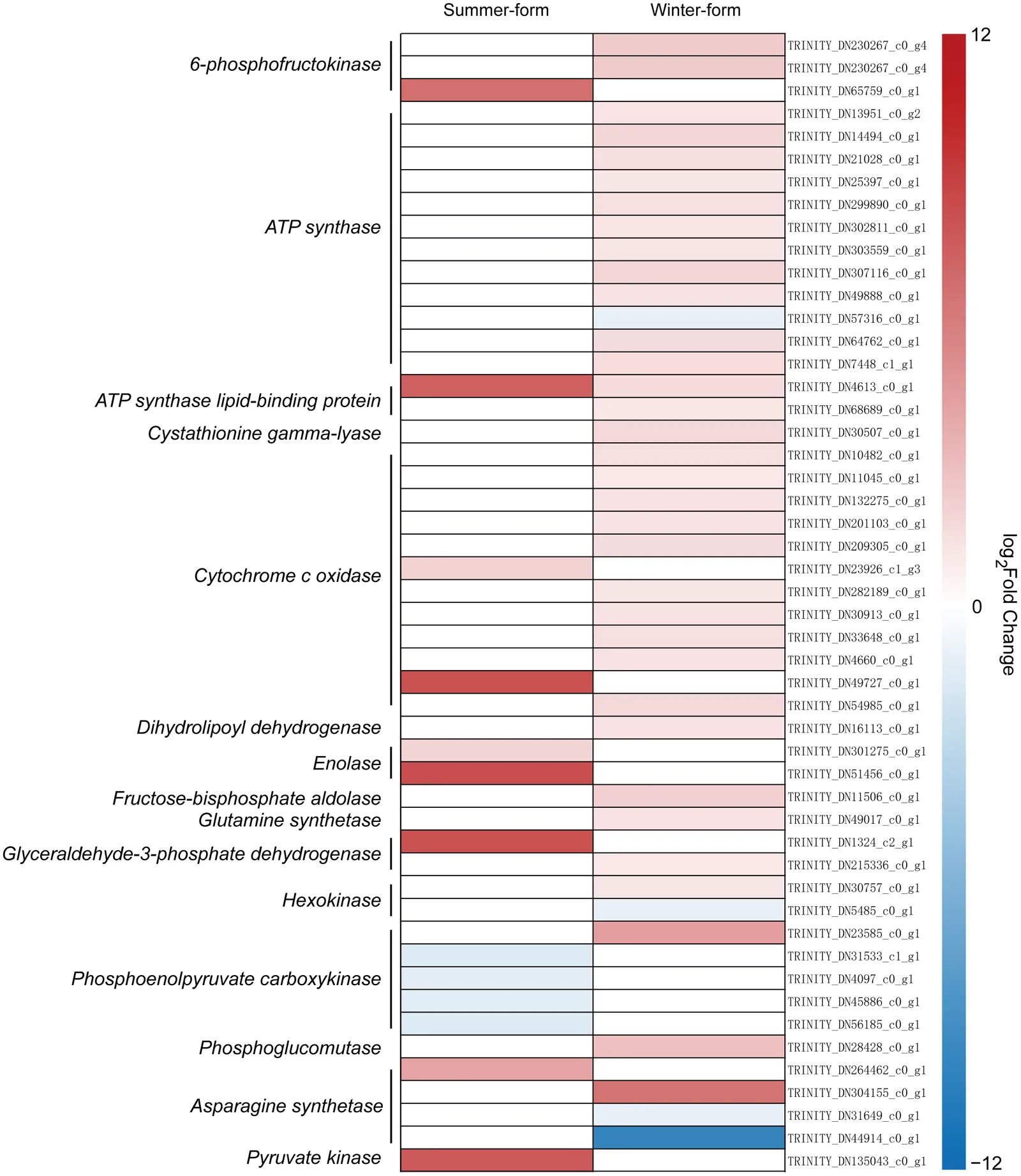
Expression heatmap of key metabolic pathway genes in summer-form and winter-form *C. chinensis* nymphs after *B. bassiana* infection. The color scale represents log_2_Fold Change, with red indicating upregulation and blue indicating downregulation.

Analysis of expression patterns of key metabolic pathway genes revealed significant differences in metabolic gene responses between summer-form and winter-form nymphs. Summer-form nymphs had fewer significantly upregulated metabolic genes, mainly including *6-phosphofructokinase*, *ATP synthase lipid-binding protein*, *dihydrolipoamide dehydrogenase*, *enolase*, *glyceraldehyde-3-phosphate dehydrogenase*, and *pyruvate kinase*. Winter-form nymphs had more significantly upregulated metabolic genes, including *ATP synthase*, *cystathionine gamma-lyase*, *cytochrome c oxidase*, *glutamine synthetase*, *hexokinase*, and *asparagine synthetase*, involving multiple metabolic pathways such as tricarboxylic acid cycle, oxidative phosphorylation, and amino acid synthesis, indicating that winter-form nymphs activated a more comprehensive energy metabolism network to provide sufficient material and energy basis for immune responses (**Figure 3**).

**Figure 4:**
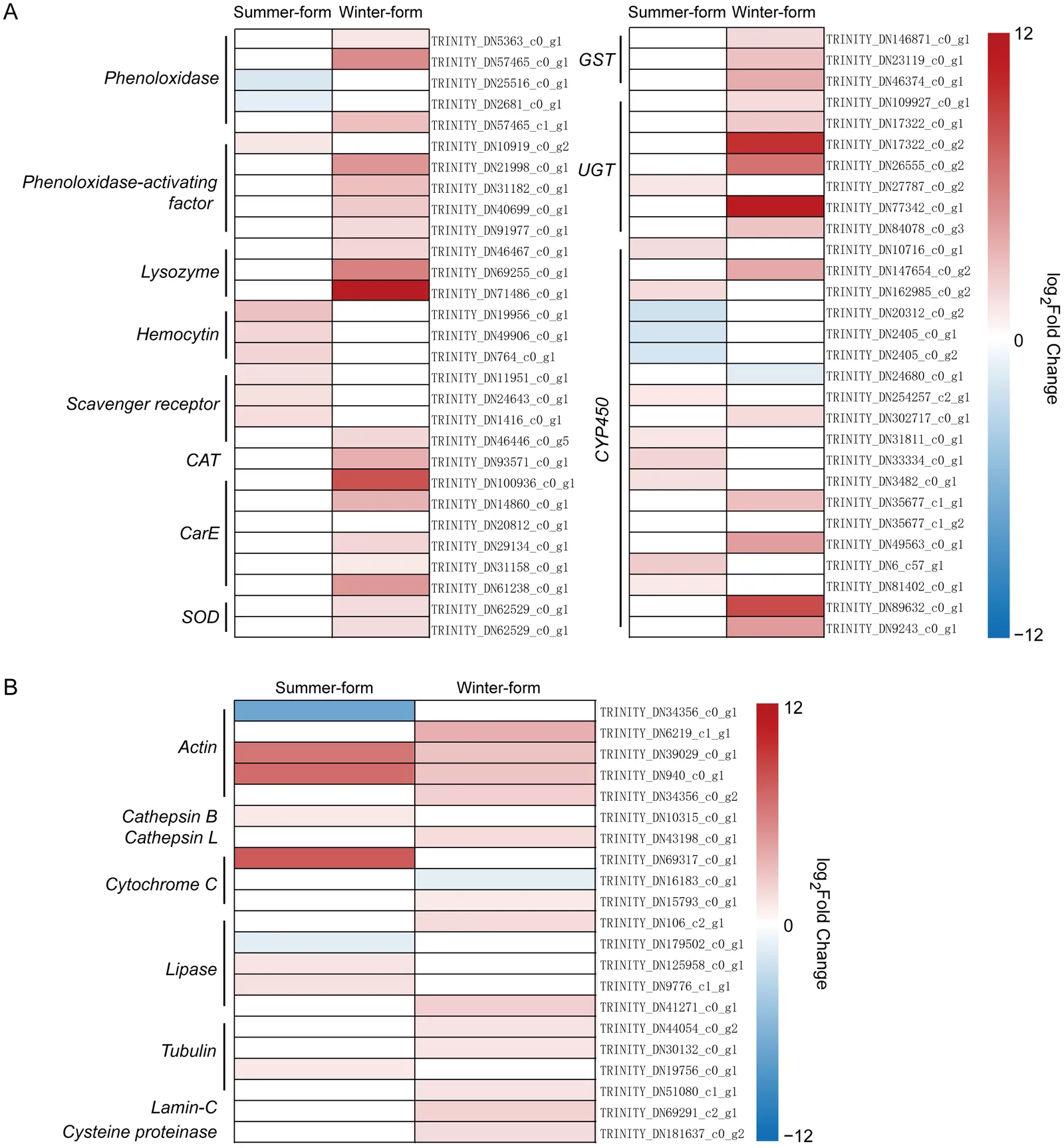
Expression heatmaps of immune-related and cell-related genes in summer-form and winter-form *C. chinensis* nymphs after *B. bassiana* infection. **(A)** Immune-related genes. **(B)** Cell-related genes. The color scale represents log_2_Fold Change, with red indicating upregulation and blue indicating downregulation.

Analysis of humoral immunity-related gene expression patterns showed that numerous immune-related genes were significantly upregulated in winter-form nymphs, including *phenoloxidase*, *phenoloxidase-activating factor*, *lysozyme*, *catalase*, *carboxylesterase*, *superoxide dismutase*, *glutathione S-transferase*, *UDP-glucuronosyltransferase*, and *cytochrome P450*. Summer-form nymphs mainly upregulated pattern recognition receptor genes such as *hemocyanin* and *scavenger receptor*, as well as two types of detoxification metabolism-related genes *UDP-glucuronosyltransferase* and *cytochrome P450*, with two *phenoloxidase* and three *cytochrome P450* genes downregulated (**Figure 4A**).

Analysis of cellular immunity-related gene expression patterns showed that genes involved in cytoskeleton and phagocytosis were generally upregulated in winter-form nymphs, including: 4 *actins*, 1 *cathepsin L*, 1 *cytochrome C*, 2 *lipases*, 3 *tubulins*, 1 *lamin C*, and 1 *cysteine protease*. Summer-form nymphs only had 2 *actins*, 1 *cathepsin B*, 1 *cytochrome C*, 2 *lipases*, and 1 *tubulin* upregulated, with 1 *actin* and 1 *tubulin* downregulated (**Figure 4B**).

### 3.5 Transcriptional and enzymatic validation of immune responses

The immune system is the first line of defense against invading pathogens. Therefore, we measured the expression of six immune-related genes and the activities of four immune-related enzymes in response to B. bassiana infection.

**Figure 5:**
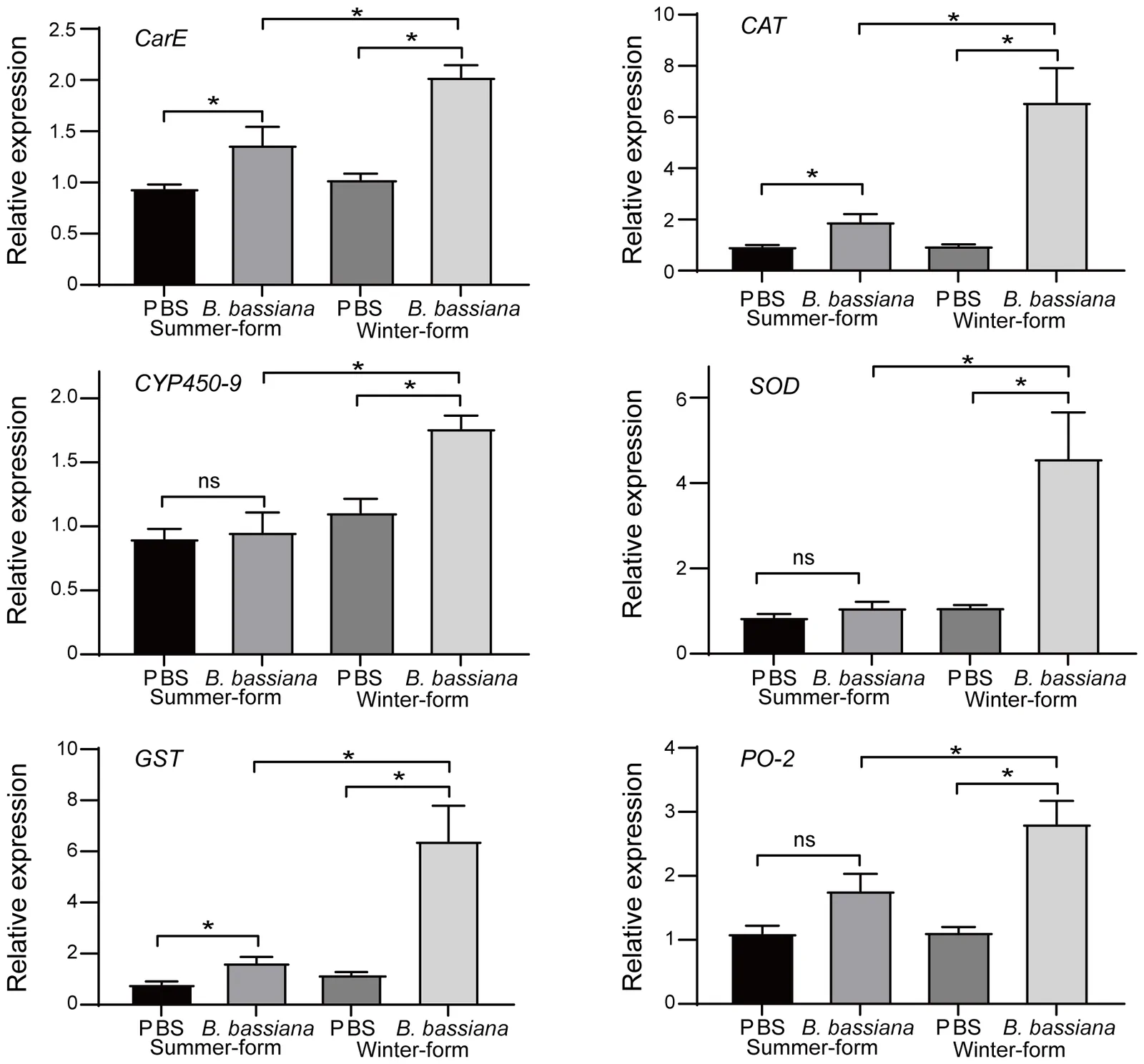
Transcriptional validation of immune-related genes in summer-form and winter-form *C. chinensis* nymphs after *B. bassiana* infection. qRT-PCR analysis of six immune-related genes at 36 h post-infection. Relative expression levels of *carboxylesterase* (*CarE*), *superoxide dismutase* (*SOD*), *cytochrome P450-9* (*CYP450-9*), *catalase* (*CAT*), *glutathione S-transferase* (*GST*), and *phenoloxidase-2* (*PO-2*). The internal reference gene *rPL45* was used for normalization.Data are presented as mean ± SEM. Asterisks indicate significant differences (unpaired two-tailed t-test: *p < 0.05); ns, not significant.

qRT-PCR analysis showed that the expression trends of all six genes were basically consistent with the RNA-seq results. In winter-form nymphs, B. bassiana infection significantly upregulated all six genes compared to the PBS control (*CarE*: *df* = 4, *p* = 0.0015; SOD: *df* = 4, *p* = 0.0322; *CYP450-9*: *df* = 4, *p* = 0.0113; *CAT*: *df* = 4, *p* = 0.0132; *GST*: *df* = 4, *p* = 0.0189; *PO-2*: *df* = 4, *p* = 0.0099). In summer-form nymphs, the response was more limited, with only *CarE* (*df* = 4, *p* = 0.0029), *CAT* (*df* = 4, *p* = 0.0299), and *GST* (*df* = 4, *p* = 0.0288) showing significant upregulation, while *SOD* (*df* = 4, *p* = 0.226), *CYP450-9* (*df* = 4, *p* = 0.7854), and *PO-2* (*df* = 4, *p* = 0.0825) showed no significant change. Although *CarE*, *CAT*, and *GST* expression was significantly induced in both morphs, the expression levels in infected winter-form nymphs were significantly higher than those in infected summer-form nymphs (*CarE*: *df* = 4, *p* = 0.0214; *CAT*: *df* = 4, *p* = 0.026; *GST*: *df* = 4, *p* = 0.0263) (**Figure 5**).

**Figure 6:**
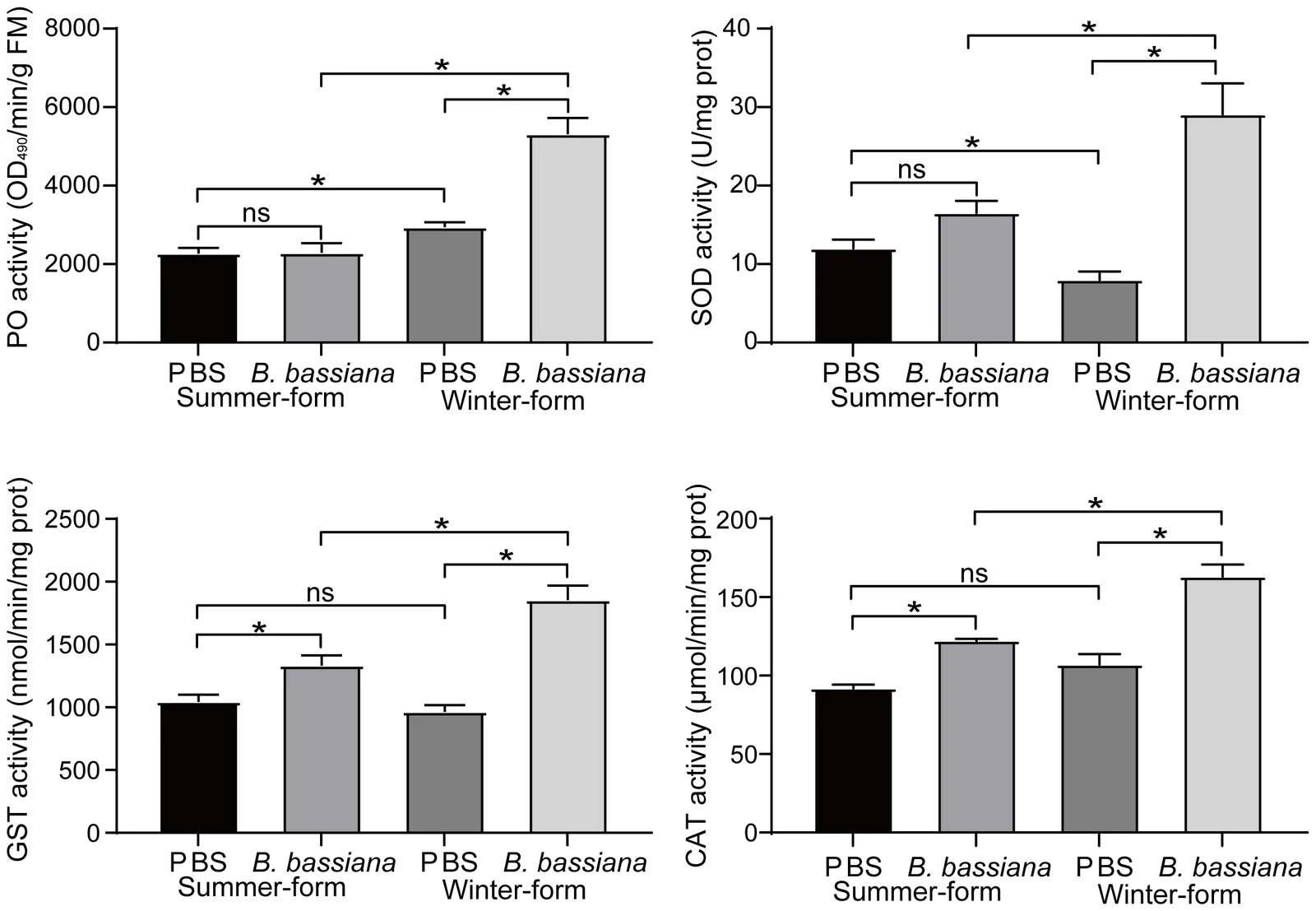
Enzymatic activity assays of immune-related enzymes in summer-form and winter-form *C. chinensis* nymphs after *B. bassiana* infection. Phenoloxidase (PO), superoxide dismutase (SOD), glutathione S-transferase (GST), and catalase (CAT) activities were measured at 36 h post-infection. Data are presented as mean ± SEM. Asterisks indicate significant differences (unpaired two-tailed t-test: *p < 0.05); ns, not significant.

Enzymatic activity assays further revealed differences between the two morphs. In winter-form nymphs, B. bassiana infection significantly induced the activities of all four enzymes: CAT (*df* = 6, *p* = 0.0016), PO (*df* = 6, *p* = 0.0015), GST (*df* = 6, *p* = 0.0004), and SOD (*df* = 6, *p* = 0.002). In summer-form nymphs, only CAT (*df* = 6, *p* < 0.0001) and GST (*df* = 6, *p* = 0.0263) activities were significantly increased, while PO (*df* = 6, *p* = 0.9426) and SOD (*df* = 6, *p* = 0.0545) showed no significant change. Although CAT and GST activities were significantly induced in both morphs, the enzyme activities in winter-form nymphs were significantly higher than those in summer-form nymphs (CAT: *df* = 6, *p* = 0.002; GST: *df* = 6, *p* = 0.0102) (**Figure 6**).

## 4 Discussion

Physiological and immune differences caused by seasonal polymorphism in insects are factors affecting the stability of biocontrol fungal efficacy. *C. chinensis*, as a typical hemipteran pest with seasonal polymorphism, has its nymphal stage as the most concentrated harmful period in its annual life cycle and a developmental stage with high control potential. The present study demonstrates that the seasonal phenotypic polymorphism of *C. chinensis* nymphs is accompanied by markedly distinct immune and metabolic responses to *B. bassiana* infection, revealing the molecular basis of stronger fungal resistance in winter-form nymphs. The research results provide theoretical basis for developing green control technologies against *C. chinensis* and targeted optimization of *Beauveria* formulations.

Seasonal polymorphism is an important adaptive strategy evolved by insects to cope with environmental fluctuations, widely distributed across insect groups and considered a core dimension of ecological adaptability (Fordyce, 2006). The present study demonstrates that the phenotypic differences between summer-form and winter-form *C. chinensis* nymphs are accompanied by markedly distinct immune and metabolic responses to *B. bassiana* infection. Bioassay results showed that *B. bassiana* exhibited significantly higher virulence against summer-form nymphs than winter-form nymphs across all tested concentrations. The LC_50_ values further quantified this difference, with summer-form nymphs showing approximately 79-fold higher sensitivity than winter-form nymphs. This morph-specific susceptibility pattern is consistent with observations reported in other insect species—for example, in *Diaphorina citri*, where non-blue morph individuals showed significantly higher sensitivity to *B. bassiana* than blue morph individuals (Hosseinzadeh et al., 2021)—and aligns with the known capacity of *B. bassiana* to infect *C. chinensis* adults (Ji et al., 2025), indicating that this differential fungal resistance is a stable, developmentally conserved trait of seasonal morphs rather than a stage-specific phenomenon, thereby further supporting a general coupling between insect phenotypic polymorphism and immune capacity. From a life-history perspective, this resistance difference aligns closely with the seasonal ecological adaptation strategy of *C. chinensis* (Schwenke et al., 2016). Winter-form nymphs occur in early spring and late autumn under low temperatures and relatively scarce food resources, facing more complex pathogenic microbial pressures; long-term evolution has likely endowed them with stronger innate immune reserves. In contrast, summer-form nymphs occur during the warm pear tree growing season, where rapid population reproduction and overlapping generations favor resource investment in growth over immunity, potentially leading to higher pathogen susceptibility. The high sensitivity of summer-form nymphs to *B. bassiana* provides an important window for implementing biological control during the pear tree growing season, offering a theoretical basis for achieving effective pest suppression while reducing chemical pesticide use. It should also be noted that *B. bassiana* efficacy is highly dependent on environmental humidity. In northern pear-growing regions with relatively dry spring conditions, field application may require supplemental irrigation or formulation optimization to ensure adequate moisture for fungal infection.

At the molecular level, the immune regulatory strategies of the two morphs showed marked divergence. Winter-form nymphs exhibited a typical “multi-pathway coordination” pattern, characterized by highly coordinated activation of cellular immunity, humoral immunity, and oxidative stress regulatory systems. At the cellular immunity level, winter-form nymphs showed widespread upregulation of genes related to cytoskeletal dynamics and lysosomal function, supporting hemocyte proliferation and motility (Schwenke et al., 2016; Sun et al., 2021; Weiss-Sadan et al., 2019). This molecular-level response corresponds closely to the hemocyte counting results—winter-form nymphs showed significantly increased total hemocyte count after infection, suggesting that their cellular immunity pathway was fully activated, thereby forming an effective pathogen clearance system. However, the increase in hemocyte count was not significantly different between the two morphs, likely because only total hemocyte counts were measured. Classification of hemocyte types might have revealed more pronounced differences. At the humoral immunity level, winter-form nymphs simultaneously activated multiple key effector pathways. Among these, phenoloxidase-activating factors and phenoloxidases—key enzymes of the melanization reaction—were significantly upregulated in winter-form nymphs, along with lysozymes, contributing to pathogen encapsulation and direct antimicrobial defense (Dolezal, 2023; Karthik et al., 2014; Sheehan et al., 2018; Veillard et al., 2016). Notably, winter-form nymphs simultaneously initiated finely tuned oxidative stress regulation mechanisms under high immune activation states. Antioxidant enzyme genes such as *CATs* and *SODs*, as well as various detoxification enzymes (like *GSTs*), showed significantly upregulated expression. These enzyme systems cooperatively eliminate excessive free radicals, peroxides, and other harmful metabolic byproducts produced during immune activation, effectively preventing oxidative damage accumulation in host tissues, thereby maintaining efficient bactericidal capability while ensuring immune system homeostasis and long-term sustainability (Scott and Wen, 2001). This coordinated balance between immune activation and oxidative stress regulation allows winter-form nymphs to maintain efficient immune defense while avoiding excessive self-damage. In contrast, summer-form nymphs exhibited deficiencies across all aspects of immune response. Hemocyte proliferation after infection was weaker than in winter-form nymphs, and the number and functional diversity of DEGs were substantially lower, suggesting generally reduced immune perception and response capacity. Particularly noteworthy was the downregulation of phenoloxidases—key genes in melanization—in summer-form nymphs, likely weakening the host’s ability to encapsulate and isolate pathogens. The combination of weak cellular immunity and suppressed melanization may underlie the high susceptibility of summer-form nymphs to fungal infection.

Immune responses are energetically costly physiological processes—from immune cell proliferation and antimicrobial peptide synthesis to phagosome formation and melanization cascade activation, all requiring sustained ATP and biosynthetic precursor supplies. Consequently, insects must redirect metabolic flux from growth and reproduction toward immune defense (Anderl et al., 2026; Dolezal et al., 2019; Luo et al., 2022). In winter-form nymphs, oxidative phosphorylation, the tricarboxylic acid (TCA) cycle, and multiple amino acid synthesis pathways were significantly enriched after infection, with key rate-limiting enzymes (*hexokinases*, *ATP synthases*, *glutamine synthetase*) generally upregulated. Oxidative phosphorylation provides efficient ATP production for energy-consuming immune processes, while enhanced TCA cycle activity supplies both reducing equivalents (NADH, FADH_2_) and signaling metabolites (citrate, α-ketoglutarate, succinate) that regulate immune response intensity and duration (Inigo et al., 2021; Martínez-Reyes and Chandel, 2020; Senior, 1988). Activation of amino acid metabolism pathways provides material substrates for cell proliferation and immune effector molecule synthesis (Scaraffia and Wells, 2003; Xu et al., 2015). This “metabolism-immunity” coordinated regulatory model is widespread in insects. In *Drosophila melanogaster*, adenosine signaling switches metabolism from energy storage to mobilization mode to support immune execution (Bajgar et al., 2015). In *Bombyx mori* and *Spodoptera litura*, baculoviruses hijack adenosine signaling to reprogram host energy metabolism for self-replication (Chang et al., 2020; Lin et al., 2020). In summer-form nymphs, however, far fewer metabolic genes were upregulated after infection. Notably, *phosphoenolpyruvate carboxykinases*—hub genes connecting gluconeogenesis with the TCA cycle—showed decreased expression, suggesting that summer-form nymphs may have a reduced capacity to mobilize energy substrates into energy production pathways (Guo et al., 2019). This metabolic suppression may further contribute to immune deficiency, potentially creating a ‘metabolic inhibition–immune deficiency’ cycle.

In summary, this study tested the hypothesis that seasonal nymph morphs of *C. chinensis* differ in susceptibility to *B. bassiana*, and demonstrated that winter-form nymphs possess markedly stronger fungal resistance than summer-form nymphs. This differential susceptibility is underpinned by divergent immune-metabolic strategies: winter-form nymphs engage a coordinated, multi-layered defense spanning cellular immunity, humoral immunity, oxidative stress regulation, and energy metabolism, whereas summer-form nymphs exhibit immune suppression and metabolic constraint. These findings provide mechanistic insight into how seasonal phenotypic plasticity shapes host–pathogen interactions in this economically important pest, and offer a theoretical basis for seasonally optimized biocontrol. It should be noted that the functional annotation of *C. chinensis* unigenes relied heavily on homology to *D. citri*, which may limit the detection of species-specific immune genes. Future studies should investigate the upstream signaling pathways governing the seasonal switch in immune phenotypes, validate the field efficacy of targeting summer-form nymphs with *B. bassiana* during the pear tree growing season, and incorporate genome sequencing of *C. chinensis* to enable more accurate functional characterization of immune-related genes.

## Data Availability

The datasets presented in this study can be found in online repositories. The names of the repository/repositories and accession number(s) can be found in the article/**Supplementary Material**.
