# Supplementary material for: Summer-form susceptible, winter-form resistant: differential susceptibility of Cacopsylla chinensis nymphs to Beauveria bassiana
Source: Front Cell Infect Microbiol. 2026 Aug 19;16:1924929. doi: 10.3389/fcimb.2026.1924929 (PMC13533655; doi:10.3389/fcimb.2026.1924929)
Supplement: Supplementary file 1 [file SupplementaryFile1.docx]

Supplementary Material

# 1 Supplementary Table and Figure

## 1.1 Supplementary Table

**Supplementary Table 1.** The primers used in the current study.

| **Primer ID** | **Forward primer(5′ to 3′)** | **Reverse primer(5′ to 3′)** |
| --- | --- | --- |
| rpL45 | CCCTGGACTTTGAACAGGAA | CTCGTGGATACCGCAAGATT |
| TRINITY_DN3304_c0_g1 | TCAACAGCCGTTATGGGGAC | GTTGCTTGCCACAATCCCTG |
| TRINITY_DN70656_c0_g1 | CTATTCAGCAACGTCGCAGC | CCTGTCCACAGCACATCCAT |
| TRINITY_DN7690_c0_g1 | GGGAGTAGGAAGGAGACGGA | TGGCTGCTGCTTTGAAGAGA |
| TRINITY_DN17420_c0_g1 | CGTGGACCTCAAAACCGGTA | CGGACGTTGGTTCTGTGGTA |
| TRINITY_DN2458_c1_g1 | ATTATCCCGCCGAAGTTCCC | CGAAGCAATGAGAATCGGGC |
| TRINITY_DN16691_c1_g2 | TCGTAACCCAGGAAGGCAAC | TCTGAAACCGGCTTCCAGTC |
| CarE | CCTGCCCATGTTCCTAACGT | ACCAGCGAGGAAATGGGAAG |
| SOD | GGACTGGCTGAAGGAAACCA | AAATGAGGCCCAGCACTTGT |
| CYP450-9 | CCCTCACCACTCCTGTGTTC | CGGTGATGAGGTCGACGTAG |
| CAT | CCTTCAGAACGCCACTGACT | GCCCTCAGTTTCTGACCGAA |
| GST | AGTACCCAAACGTGGCCAAA | CTTTGAAGGCCTGGAGTCCC |
| Phenoloxidase-2 | AGACTTGGACAGATGGCGTG | TCGCCATACAGTGCCTTGTT |

**Supplementary Table 2.** Summary of transcriptome sequencing data for the 12 samples used in this study.

| **Sample ID** | **Treatment** | **Clean Reads** | **Clean Base (Gb)** | **Q30 (%)** |
| --- | --- | --- | --- | --- |
| SumP-1 | Summer-form + PBS | 26,409,309 | 7.89 | 93.36 |
| SumP-2 | Summer-form + PBS | 24,101,855 | 7.21 | 92.55 |
| SumP-3 | Summer-form + PBS | 24,171,066 | 7.22 | 92.50 |
| SumB-1 | Summer-form + *B. bassiana* | 21,719,165 | 6.50 | 92.72 |
| SumB-2 | Summer-form + *B. bassiana* | 23,909,430 | 7.15 | 93.66 |
| SumB-3 | Summer-form + *B. bassiana* | 23,269,072 | 6.96 | 93.43 |
| WinP-1 | Winter-form + PBS | 25,919,797 | 7.75 | 92.74 |
| WinP-2 | Winter-form + PBS | 25,800,603 | 7.72 | 92.52 |
| WinP-3 | Winter-form + PBS | 24,173,702 | 7.23 | 92.35 |
| WinB-1 | Winter-form + *B. bassiana* | 23,111,044 | 6.91 | 93.24 |
| WinB-2 | Winter-form + *B. bassiana* | 25,450,204 | 7.60 | 92.75 |
| WinB-3 | Winter-form + *B. bassiana* | 23,382,101 | 6.99 | 92.60 |

## 1.2 Supplementary Figure

**Supplementary Figure 1**. Overview of transcriptome data of *C. chinensis* nymphs after *B. bassiana* infection. (**A**) Venn diagram showing the number of unigenes annotated in eight databases (NR, Swiss-Prot, KEGG, COG, KOG, eggNOG, Pfam, and GO). (**B**) Homologous species distribution of top BLAST hits based on the NR database annotation. (**C**) Number of DEGs in summer-form and winter-form nymphs after *B. bassiana* infection.

**Supplementary Figure 2.** Validation of gene expression levels. RNA-Seq data are shown as the mean of three replicates, qRT-PCR data are shown as mean±SE (*n* = 3).

**Supplementary Figure 3.** GO classification of DEGs in the two seasonal morphs of *C. chinensis*.

**Supplementary Figure 4.** KEGG pathway classification of DEGs in the two seasonal morphs of *C. chinensis*.
